# 3D Multi-isotope Imaging Mass Spectrometry Reveals Penetration of ^18^O-Trehalose in Mouse Sperm Nucleus

**DOI:** 10.1371/journal.pone.0042267

**Published:** 2012-08-27

**Authors:** Claude P. Lechene, Gloria Y. Lee, J. Collin Poczatek, Mehmet Toner, John D. Biggers

**Affiliations:** 1 National Resource for Imaging Mass Spectrometry, Division of Genetics, Harvard Medical School, Brigham and Women's Hospital, Cambridge, Massachusetts, United States of America; 2 Harvard Medical School, Biomedical Engineering, Massachusetts General Hospital, Boston, Massachusetts, United States of America; 3 Department of Cell Biology, Harvard Medical School, Boston, Massachusetts, United States of America; National Cancer Institute, United States of America

## Abstract

The prevalence of genetically engineered mice in medical research has led to ever increasing storage costs. Trehalose has a significant beneficial effect in preserving the developmental potential of mouse sperm following partial desiccation and storage at temperatures above freezing. Using multi-isotope imaging mass spectrometry, we are able to image and measure trehalose in individual spermatozoa. We provide the first evidence that trehalose penetrates the nucleus of a mammalian cell, permitting tolerance to desiccation. These results have broad implications for long-term storage of mammalian cells.

## Introduction

The common use of a wide variety of genetically engineered mice in medical research has led to ever increasing costs related to maintaining colonies of adult individuals required for the continuation of each line [1]. These costs could be dramatically reduced by using mouse sperm dried at ambient temperature and stored in a glassy state at non-cryogenic temperature. The technique requires very simple equipment, can be completed in a short time (minutes), and does not require the use of liquid nitrogen [2], [3], giving it significant advantages over the alternative techniques of cryopreservation and freeze-drying [4]. In nature, many anhydrobiotic plants and animals living in dry or polar conditions synthesize trehalose from glucose to facilitate the creation of a glassy state that protects against deterioration during periods of desiccation [5]. Mammalian cells do not have the metabolic pathways needed to synthesize trehalose, nor are their cell membranes permeable to this disaccharide. Thus to preserve mammalian cells using trehalose, the compound has to be artificially introduced in high concentrations and distributed throughout the cell (including the nucleus). By first porating the sperm cells with α-hemolysin, we were able to introduce trehalose molecules. The extent to which trehalose actually penetrates the sperm nucleus, which contains densely-packed chromatin, is not known. But by labeling the trehalose with ^18^O and using multi-isotope imaging mass spectrometry (MIMS) and quantitative MIMS tomography (QMT), we have conclusively shown that trehalose penetrates the nucleus of individual sperm and thus can contribute to maintaining a glassy state around the chromatin and preserving DNA without using liquid nitrogen. MIMS is based on secondary ion mass spectrometry (SIMS). SIMS is one of the most sensitive ands precise analytical methods in existence and is one of the staple methodologies in cosmochemistry, geochemistry, material sciences, and paleo-dating. We are developing MIMS for precise metabolic measurements of stable isotope tagged molecules in intracellular volume smaller than 50 nm^3^ [6], [7], [8], [9].

## Results

**Figure 1:**
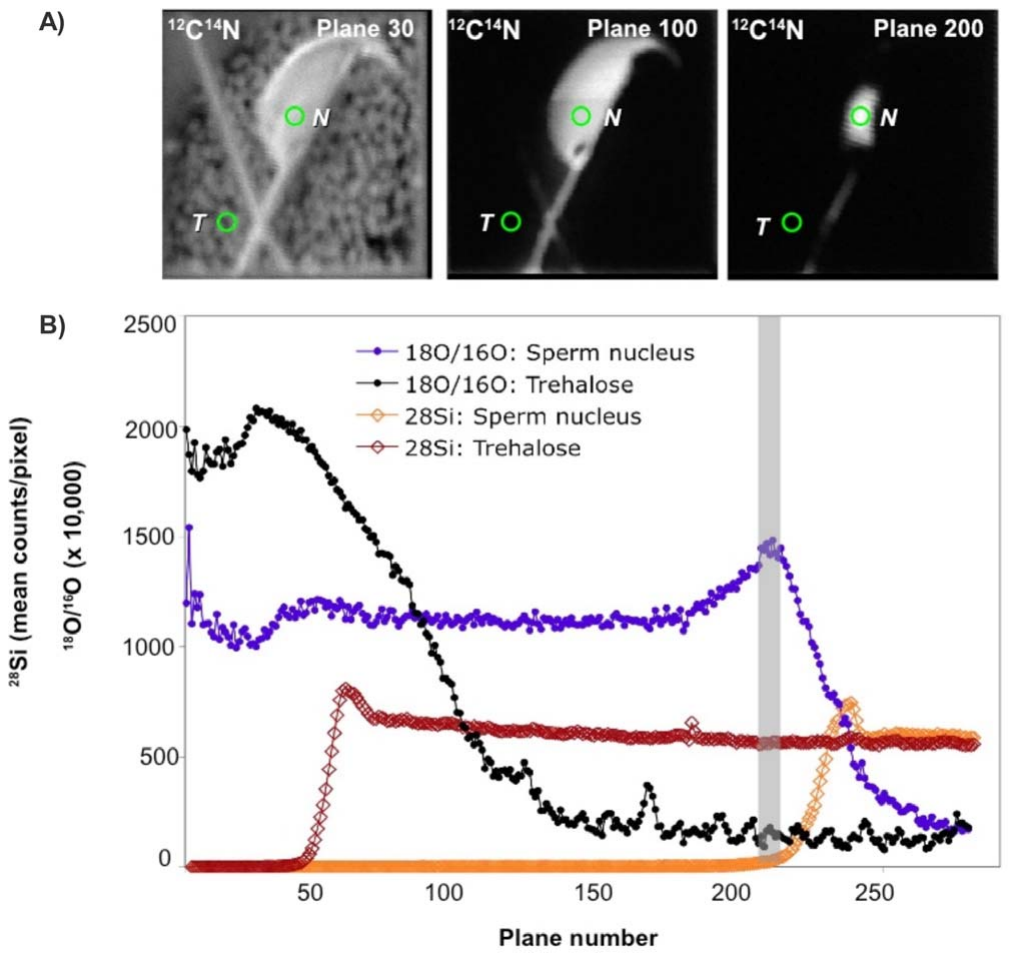
MIMS images from 3 planes and data from a full-thickness study. **A**) ^12^C^14^N quantitative atomic mass images of a mouse sperm head extracted from planes 30, 100 and 200 out of 284 (image field = 15 µm×15 µm; 256×256 pixels; acquisition time per plane = 5.5 mins; total acquisition time = 27 hrs). Green circles: ROIs in sperm head (“N”) and in trehalose film (“T”) used to generate plots of ^18^O/^16^O values and ^28^Si counts. **B**) ^18^O/^16^O ratios (circles) and ^28^Si counts (diamonds) extracted from each plane from the ROIs shown in (A). The spikes in the ^28^Si curves show the point at which the trehalose film (red diamonds) and the sperm head (orange diamonds) have been completely sputtered through. The vertical grey stripe indicates end of sperm head sputtering and the start of silicon exposure.

We analyzed individual sperm samples using MIMS methodology, acquiring parallel quantitative mass images for ^16^O, ^18^O, ^12^C^14^N and ^28^Si. A whole sperm was analyzed using quantitative MIMS tomography (QMT) by acquiring a stack of hundreds of image planes through the full thickness of the cell. The ^12^C^14^N quantitative atomic mass images from plane 30, plane 100 and plane 200 are shown in Fig. 1A. The image at plane 200 shows that the sperm head perimeter has decreased and that the ^12^C^14^N signal outside the sperm head is extinct. Fig. 1B shows the ^18^O/^16^O values and ^28^Si counts extracted from each plane from the regions of interest (ROIs) shown. We observe elevated ^18^O/^16^O values in the sperm head (blue circles) even after the ratio from the trehalose film (black circles) falls to the background level, conclusively demonstrating the penetration of trehalose into the sperm. The spikes in the ^28^Si curves show the points at which the trehalose film and the sperm head have been completely sputtered through. We obtained equivalent results with 9 other spermatozoa. The Movie S1 is made of the successive image planes acquired during the analysis of a sperm head.

## Discussion

The chromatin in mature spermatozoa is embedded in the nuclear matrix and consists of tightly packed DNA in toroidal structures associated with protamines, the DNA-binding proteins [10]. The sperm nucleus contains 64–69% water [11], some of which hydrates the chromatin forming a shell of water necessary for transcription and replication around the DNA [12]; the remainder either hydrates other macromolecules in the nuclear matrix or is free. Our results provide the first evidence that trehalose can freely enter the compartments of the sperm nucleus, where it can replace water molecules in the creation of a glassy state and thereby bestow tolerance to desiccation. These results have important implications in long-term storage of mammalian cells in a dry state at ambient conditions.

α-hemolysin creates pores in cell membranes from 1.4 to 4.6 nm diameter [13]. These pores are large enough to permit the passive diffusion of trehalose, whose molecular diameter with a single hydration sphere has been estimated to be 1.2 to 1.3 nm [14]. In contrast, the α-hemolysin molecule, which has a molecular mass of 33.2 kD, cannot pass into the cell to damage the nuclear membrane. A slight chemical modification of α-hemolysin can increase the permeability of mouse fibroblasts to sucrose, a molecule with a size similar to trehalose [15]. Small molecules can enter the nuclear matrix by passive diffusion through nuclear pore complexes, which are scattered throughout nuclear membranes. In Hela cells, the complexes contain pores whose diameters vary around a mean of ∼5.0 nm [16]. The nuclear pore complexes, however, are not distributed all over the nuclear membrane of a mature spermatozoon in many mammals. Instead, during the final stage of spermatogenesis when the spermatid transforms into the mature spermatozoon, the volume of the nucleus is greatly reduced, and the surplus nuclear membrane folds to form the redundant nuclear envelope [17]. During this process in the mouse, the nuclear pore complexes become exclusively localized in the redundant nuclear envelope at the caudal end of the nucleus [18]. Presumably, trehalose enters the sperm nucleus at the caudal end of the nucleus from where it is distributed throughout the nuclear matrix.

One reason a plasticizer, such as trehalose, is introduced into the sperm is to increase the temperature of the glassy state (T_g_) so that sperm can be stored at a higher, more convenient temperature. More recent work on evaporative drying has shown that mouse sperm will survive and retain their fertilizing ability after storage slightly above the T_g_ in a highly viscous rubbery state, suggesting that the extremely condensed chromatin is naturally in a glassy or in a close-to-glassy state [2], [3], [19], [20].

## Materials and Methods

### Sperm Donors

Three- to nine-months-old B6D2F1 male mice were used as sperm donors. All procedures involving these mice have been reviewed and approved by the Massachusetts General Hospital Subcommittee on Research Animal Care (No. A 3596-01). Mice were maintained in accordance with guidelines of the Committee on Care and Use of Laboratory Animal Resources, National Research Council.

### Reagents and Media

All reagents were obtained from Sigma Chemical Co. (St. Louis, MO) unless otherwise noted. The sperm isolation medium was prepared as outlined elsewhere [21]. Briefly, this EGTA medium contained 10 mmol/L Tris-HCL buffer and 50 mmol/L each of NaCl and EGTA, adjusted to pH of 8.3 with KOH. Poration stock medium of 25-mg/ml α-hemolysin in HBS1 and trehalose loading medium of 1.0-mmol/L trehalose in EGTA medium were prepared as described previously [2].

### Sample Preparation

Sperm sample preparation followed the protocol described in detail by McGinnis et al [2]. Essentially, the male mice were anesthetized with isoflurane USP (Abbott Laboratories, Chicago, IL). Caudal epididymides were excised, placed in 1 ml of EGTA medium, and cut in several places to release the sperm. The sperm suspension was transferred into a 1.5 ml conical tube and allowed live sperm swim-up for 15 min. at 37°C. From the top portion of the sperm suspension column, 100 µl was transferred to another conical tube containing 100 µl α-hemolysin stock medium to allow poration for 15 min at room temperature. After poration, 200 ml EGTA-trehalose stock medium was added and incubated for 30 minutes at room temperature. The final concentration of trehalose in the sperm sample was 0.5 M.

**Figure 2:**
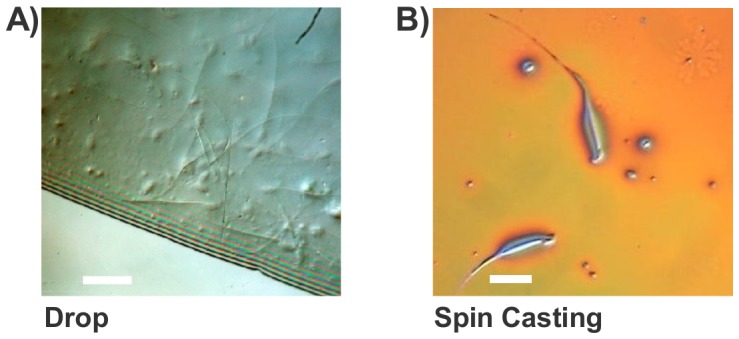
Reflection differential interference micrographs of two preparation methods. **A**) Mouse sperm deposited drop-wise onto a silicon chip. **B**) Sperm prepared with spin casting. Bar is 50 µm.

Our initial sperm preparation methods resulted in sperm samples buried in a thick caramel-like layer of the 0.5-M trehalose solution (Fig. 2a), which were not suitable for MIMS analysis. A better preparation method is to spin dry the sperm/trehalose solution [22], [23], producing a thin layer of trehalose containing evenly distributed, non-overlapping sperm (Fig. 2b). Spin cast trehalose-sperm were prepared using a Headway Research spinner. A 5-mm×5-mm Si chip was secured to the spinner's vacuum chuck, and the chip was spun at 3500 rpm. A total of 100 µl of the 0.5-M trehalose-sperm solution was deposited in 10-µl increments at 10-second intervals. This allowed ample time for the trehalose to spread across the Si chip and dehydrate, forming a thin, glassy film. The films were measured with a Dektak IIA surface profilometer from Sloan Technology Corp., operating at medium speed with a total scan length of 700 µm. Films varied in thickness from 300 nm–600 nm. Using this sample preparation method, we have demonstrated the penetration of trehalose in sperm incubated with ^18^O-trehalose (synthesized by Unkefer at the late Stable Isotope Laboratory, LANL).

### MIMS Data Acquisition

The factory prototype of the NanoSIMS50 (Cameca, Courbevoie, France) was used for MIMS analysis as previously described [7]. A focused beam of Cs+ ions was used to sputter and ionize secondary ions from the samples. The Cs+ primary ions were scanned over a raster pattern of either 256×256 pixels or 512×512 pixels, to analyze individual sperm. At each pixel location, the secondary ion intensities for for ^16^O, ^18^O, ^12^C^14^N and ^28^Si. were recorded in parallel from the same sputtered volume. We analyzed entire spermatazoa by acquiring large stacks of image planes.

### Data Reduction and Analysis

From the acquired full-thickness image stacks, we extracted the four original quantitative mass images (QMIs; ^16^O, ^18^O, ^12^C^14^N and ^28^Si). The ratio image (^18^O/^16^O) was derived by the pixel-by-pixel division of the ^18^O QMI by the ^16^O QMI. We selected regions of interest (ROIs) indicating the nucleus and the trehalose. ROIs were then projected through the image stacks on all QMI and ratio images to extract quantitative data. Statistics for each ROI were then tabulated using custom applications run within ISee software (Inovision Corporation, Raleigh, NC). The data were further reduced using the spreadsheet, statistical, graphing and modeling program JMP6 (SAS Institute, Cary, NC).

## Supporting Information

Movie S1 **Movie showing the depth profiles of the ratio ^18^O/^16^O, ^28^Si and ^14^N (^12^C^14^N) over regions of interest locates on the sperm head (blue circle) and on the support (silicon covered with trehalose, red circle).**
(M4V)
